# Growth Performance, Blood Metabolites, Carcass Characteristics and Meat Quality of Lambs Fed Diets Containing Different Energy Levels Supplemented with Rumen-Protected Choline

**DOI:** 10.3390/ani14111682

**Published:** 2024-06-05

**Authors:** Salih Çelik, Habip Muruz

**Affiliations:** 1 The Ministry of Agriculture and Forestry of the Republic of Turkey, Tokat Provincial Office, İmamlık Street. No:68, Tokat 60200, Turkey; celik.salih@tarimorman.gov.tr; 2 Department of Animal Nutrition and Nutritional Diseases, Faculty of Veterinary Medicine, Ondokuz Mayıs University, Kurupelit Kampusu, Samsun 55100, Turkey

**Keywords:** rumen-protected choline, lamb, energy, performance, meat

## Abstract

**Simple Summary:**

Simple Summary: Supplementation with rumen-protected choline (RPC) can reduce dietary metabolizable energy (ME) while maintaining optimum performance, thus contributing to reducing production costs by saving grain which is the primary source of energy in the diet. Previous papers have reported on the interaction effects of ME level and RPC supplementation on production outcomes in dairy cattle and growing goats. The objective of this study was to examine the effects of ME level and RPC supplementation on the growth performance, carcass characteristics, meat quality, serum energy, lipid, and protein profiles of Karayaka lambs. In conclusion, the supplementation of RPC at 0 and 5 g/kg did not affect lamb growth performance and carcass characteristics. Given the lack of significant improvements in performance metrics, RPC supplementation would not result in net cost savings. However, it may play a role in modulating nitrogen metabolism under different dietary energy conditions, as indicated by the significant reduction in serum urea-N levels. The results of the current study indicate that there is no benefit to be gained from the supplementation of RPC in the low-energy diet of fattening lambs.

**Abstract:**

This study aimed to examine the effects of metabolizable energy (ME) level and rumen-protected choline (RPC) supplementation on the growth performance, carcass characteristics, meat quality, serum energy, lipid, and protein profiles of Karayaka lambs. Twenty-eight Karayaka lambs, with an initial body weight (BW) of 26.85 ± 0.26 kg, were randomly assigned (2 × 2 factorial design) to one of four dietary treatments with two levels of ME (optimum: 2750 or low: 2500 kcal ME/kg dry matter) and two levels of RPC (0 or 4 g/d/lamb). Lambs of each group were housed in individual pens. The experiment lasted 66 d, with the first 10 d consisting of acclimation and the next 56 d of the formal experimental period. The data on BW, dietary matter intake (DMI), and serum glucose concentrations confirm that our model successfully induced low energy using 250 kcal/kg less energy than the optimum level. RPC supplementation did not significantly affect average daily DMI, total average daily gain (ADG), or feed conversion ratio (FCR) at any energy level. Additionally, there was no substantial effect on carcass characteristics, meat quality, serum lipids, energy metabolism indicators, and liver function parameters. There was also no interaction effect of RPC × ME on the parameters tested. However, at 56 d into the experiment, the interaction effect of RPC × ME on serum urea-N was highly significant, and RPC supplementation led to lower serum urea-N levels (*p* = 0.001). These results suggest that while RPC supplementation did not enhance overall performance and carcass characteristics in Karayaka lambs, it may play a role in modulating nitrogen metabolism, as indicated by the significant reduction in serum urea-N levels.

## 1. Introduction

Choline (2-hydroxyethyl trimethyl ammonium salt), an essential nutrient for humans and animals, needs to be obtained through diet and endogenous synthesis. Choline plays a pivotal role in maintaining and promoting cell growth throughout life, including neurotransmission, membrane synthesis, lipid transport, and one-carbon metabolism [1]. In the liver, choline can be utilized to produce phosphatidylcholine via the cytidine diphosphate–choline pathway. Furthermore, choline can be oxidized to form betaine, which, via the phosphatidylethanolamine N-methyl transfer pathway, is involved in the synthesis of very low-density lipoprotein for lipid transport [2]. Choline can be obtained from the diet or synthesized de novo, which has made it difficult to determine the precise choline requirements in ruminants [3,4,5]. In lambs, extrahepatic choline synthesis and biliary choline re-utilization between the gut and liver contribute to maintaining a greater body reserve of endogenous choline [6]. The rumen microbiota quickly breaks down the naturally present choline in feed. The N-methyl groups are transformed into trimethylamine, which is then converted into methane [7]. Therefore, even feedstuffs rich in choline can only slightly contribute to the post-ruminal supply of choline. As a result, choline supplementation should be provided as rumen-protected choline (RPC) for ruminant animal species [8]. RPC is a commercial product designed to deliver choline to the small intestine for absorption by protecting choline chloride from ruminal degradation [9]. Experiments with rumen-protected choline (RPC) have demonstrated that choline is a limiting nutrient for domestic ruminants, mainly when the genetic potential has increased metabolic nutrient demands [10]. Previous studies reported that RPC supported final body weight (FBW) [11] and carcass characteristics [12] in finishing beef steers and improved growth performance and meat quality in finishing lambs [13,14,15]. RPC also improved nutrient digestibility, feed efficiency, milk production, and milk quality in dairy cows [16] and increased productive performance in growing goats [17]. Although the mechanism of action of choline on growth performance and carcass traits is not fully understood, it has been reported that it may be related to lipid metabolism [12,13,14,15,16,17,18,19], protein metabolism [20,21], induction of slow energy metabolism [15], or modulation of hepatic gluconeogenesis [19].

Grains are a costly part of lamb diets, and it is considered a significant portion of the total cost of producing fattening lamb. Moreover, producers face challenges due to increasing grain prices worldwide and competition from sectors like biofuel production or human consumption [22]. This can result in the use of high-fiber, low-energy feeds during lamb fattening, which may negatively impact growth performance, feed efficiency, and meat quality [23]. It is widely acknowledged that dietary energy concentration can be achieved by supplementing the diet with fat or fat-rich feeds. However, this is restricted by animal metabolic physiology. Therefore, in recent years, attempts have been made to implement nutritional interventions that maintain optimum performance by reducing dietary energy concentrations, thereby reducing the total cost of production. A recent study demonstrated that the interaction of maintenance energy (1.40 Mcal NEL/kg) and RPC supplementation supported hepatic glucose and lipid metabolism through the expression of genes related to glucose and lipid metabolism, thus improving production compared to excessive energy (1.63 Mcal NEL/kg) in periparturient dairy cows [19]. In growing goats, Tu et al. [24] indicated that RPC has a greater effect on growth performance when supplemented in a reduced-energy matrix value basal diet (minus 250 kcal/kg). However, no literature on the energy matrix value of RPC in lamb diets is available. We hypothesized that dietary choline would enhance productive performance and meat quality by influencing energy and protein metabolism in lambs fed low-energy diets. Therefore, this study aimed to evaluate the effect of dietary RPC on the growth performance, carcass characteristics, meat quality, and serum biochemical indicators of Karayaka lambs fed diets containing different levels of ME viz. optimum (2750 kcal ME/kg) and low (2500 kcal ME/kg).

## 2. Materials and Methods

### 2.1. Animal Care

The animal study protocol was approved by the animal ethics committee of Ondokuz Mayıs University (protocol number 2021/46 and date of approval: 11 November 2021).

### 2.2. Animals and Design

Twenty-eight male Karayaka breed lambs, aged 120 days with an initial body weight (IBW) of 26.85 ± 0.26 kg, were obtained from a private farm in Tokat province, Turkey. The Karayaka sheep is a native breed that is intensively bred in the Black Sea region of Turkey. The breed is well-adapted to harsh environments, such as poor climates or inadequate pastures [25]. The lambs were vaccinated against clostridial infections using a mixed vaccine and dewormed against external and internal parasites using ivermectin. The lambs were grouped (7 per group) by body weight (BW), and lambs of each group were housed in individual pens (2 m × 1.5 m) equipped with feeding and automatic drinking devices. Animals were randomly assigned (2 × 2 factorial design) to one of four dietary treatments with two levels of ME (optimum: 2750 or low: 2500 kcal ME/kg dry matter) and two levels of RPC (0 or 4 g/d/lamb). The IBW of lambs was considered in randomization. 

As planned, optimum ME diets were formulated to be isocaloric (2750 kcal ME/kg) and isonitrogenic (16% CP) to meet all nutrient requirements for growing lambs (NRC, 2007), but the lower ME diet contained lower energy (2500 kcal/kg) than the optimum ME diet (Table 1). The ratio of ingredients in low- and optimum-energy concentrate feeds was determined, taking into account the restriction imposed by the applied energy reduction. Lambs were provided with ad libitum access to both concentrate feed and fresh water throughout the study. Additionally, they received a daily supply of alfalfa hay, cut into 4 cm lengths, at a rate of 150 g per lamb. The concentrate feed and forage were presented in separate feeders. Fresh feeds were provided twice daily at 8:00 and 16:30 h. The concentrate feed offered to each lamb was adjusted to minimize waste (10% of the previous day’s intake, as-fed). Feeders were visually assessed between 07:30 and 07:45 h each morning, and any remaining feed was collected and weighed for determination of dry matter intake (DMI). The level of RPC supplementation was determined based on the recommendation of Barcena-Gama et al. [26]. The RPC, which contains 25% choline chloride, was obtained from Bintaş Danışmanlık Sanayi ve Ticaret A.Ş (Tekirdağ, Türkiye). Prior to the morning feeding, the RPC was manually incorporated into a small portion of the concentrate feed to ensure that the lambs consumed the entire dose. Prior to the morning feeding, the lambs were individually weighed at the beginning of the experiment (IBW), at intermediate points (14, 28, and 42 d), and at the end of the experiment (56 d). The experiment was conducted for a total of 66 days, including a 10-day adaptation period.

### 2.3. Growth Performance Calculus

Based on the data collected during the feeding trial, the following parameters were calculated: (1) average DMI = (feed offered − feed refused), which was weighed and recorded daily; (2) ADG = [(FBW − IBW)/number of d on feed]; and feed conversion ratio (FCR) = (DMI/ADG) [27].

### 2.4. Carcass Characteristics and Sampling

At the end of the trial, the animals were weighed following a 24 h fast and with free access to water until approximately 2 h before slaughter. The Regulation on Special Rules for Official Control of Animal Foods of the Turkish Ministry of Agriculture and Forestry and standard abattoir procedures then slaughtered the animals. The hot carcass weight was immediately recorded, and the hot dressing percentage was calculated as the ratio of the hot carcass weight to the slaughter body weight. The carcasses were then chilled at 4 °C for 24 h. Afterward, they were reweighed to determine the cold carcass weight. The cold dressing percentage was calculated as the ratio of cold carcass weight to slaughter BW. To determine the area of the longissimus dorsi (LD) meat muscle in the left half of the carcass, a cut was made between the 12th and 13th ribs to expose the muscle [27]. The area was then outlined on tracing paper and measured using a digital planimeter. Fat thickness over LD was determined via measuring with a digital caliper. To assess meat quality, the meat muscle of the left LD was removed from each carcass, and the samples were stored at −20 °C until analyzed.

### 2.5. Meat Quality

For pH, meat samples were measured directly in triplicate on the LD meat muscle using a digital pH meter (Testo 205, Testo AG, Lenzkirch, Germany) 24 h postmortem. The pH probe was calibrated beforehand with pH 4.0 and 7.0 standard buffers. Meat color was determined at the three measurements taken 24 h postmortem using a CR-400 chromameter (Konica Minolta Inc., Tokyo, Japan), and the results were described as lightness (L*), redness (a*) and yellowness (b*) [28].

The meat samples were thawed at 4 °C for 24 h. The water-holding capacity of the LD muscle was measured using the filter paper press method developed by Grau and Hamm [29]. A digital precision scale was used to weigh 3 g of the sample, which was then placed between two filter papers and subjected to pressure for 5 min using a 5.0 kg weight. The meat piece was placed between two glass plates during the process. After five minutes, the weight was removed, and the meat between the two filter papers was reweighed.

The drip loss percentage was measured using the method outlined by Huo et al. [30]. About 25 g of sample from the LD meat muscle was placed in a polyethylene bag, which was inflated to prevent the sample from contacting the sides of the bag. The bag was then hung on hooks in a refrigerator at 4 °C. After 24 h, the samples were re-weighed to calculate the weight change. 

The cooking loss percentage was measured using the method described by Honikel [31]. Transverse slices of the analyzed muscles, weighing 50–70 g and with a thickness of 3 cm, were placed in thin polyethylene bags. The bags were tightly attached to the meat sample and then placed in a water bath at 70 °C for 1 h. After cooling for 30 min using running tap water, the samples were re-weighed after excess moisture was removed with paper towels. The change in weight of the sample was calculated as a percentage. 

Warner–Bratzler shear force measurements were performed using a Warner–Bratzler V-shaped blade attached to an Instron 3343 Texture Analyzer (Instron,. Norwood, MA, USA)) (50 kg load cell; test speed 2 mm/s; distance 20 mm; trigger force 0.049 N). The analysis was conducted on samples used for cooking loss and kept at 4 °C under refrigerated conditions (24 h after cooking). Before analysis, muscle samples were brought to room temperature, and six cores (1.6 cm in diameter) were extracted from each sample using a round blade parallel to the muscle fibers [32].

### 2.6. Blood Sample Collection and Preparation for Analysis of Serum Metabolite Profile

Glucose, lipid, and protein profiles and liver function tests were measured in blood samples collected on day 0, 14, 28, 42, and 56 of the experiment. Samples were collected from 7 lambs of each group via the jugular vein into a 5-mL vacuum tube without anticoagulant before the morning feeding. The obtained samples were kept at room temperature for 30 min, then centrifuged at 3000× *g* (LC-04B centrifuge, Zenith Lab, Jintan, China) for 10 min at 4 °C [28]. The serum obtained was stored at −20 °C until the analysis. Samples were analyzed for concentration of glucose and lipids profile [TG (triglycerides), cholesterol (CHOL), low-density lipoprotein (LDL), and high-density lipoprotein (HDL)], proteins profile [total protein (TP), albumin (ALB), creatinine (Cre) and serum urea-N], and liver function tests [aspartate aminotransferase (AST), alkaline phosphatase (ALP), alanine aminotransferase (ALT), gamma-glutamyltransferase (GGT), total bilirubin (TBIL)] using commercial kits in accordance with manufacturer instructions on the DRI-CHEM NX500 (Fujifil Co., Tokyo, Japan) and BS-400 (Mindray Bio-Medical Electronics Co. Shenzhen, China) Automatic Biochemical Analyzers. Globulin (GLB) concentrations were calculated as TP concentration minus ALB concentration.

### 2.7. Feed Analysis

Feed samples were analyzed for dry matter (DM), crude protein (CP), ether extract (EE), and crude cellulose according to AOAC Protocols [33].

### 2.8. Statistical Analysis

The data were analyzed using analysis of variance (ANOVA) as a 2 × 2 factorial arrangement (2 levels of RPC × 2 levels of ME). Animals were considered as experimental units. The model was designed to analyze the main effects (RPC and ME) and the interaction (RPC × ME). Shapiro–Wilk and Levene tests were used to check for normality and homogeneity of variances, respectively. All analyses were conducted using the general linear model (GLM) procedure of IBM SPSS (Version 12.0; IBM, Armonk, NY, USA). The *p*-values for significant interactions (RPC × ME) were re-analyzed using one-way ANOVA for multiple comparisons with a confidence level of *p* < 0.05.

## 3. Results

Table 1 presents the percentage composition of the two experimental concentrate feeds and alfalfa hay formulated for lambs and the nutrient calculation analysis. The calculated values for the two ME levels (2750 and 2500 kcal/kg) for RPC-free and RPC-supplemented concentrate feeds were as expected and appropriate for testing our hypothesis. The mixed feeds achieved the same CP level as designed.

### 3.1. Growth Performance

Despite consuming more feed, lambs on the low-energy diet exhibited significantly lower body weight gains compared to those on the optimal energy diet, resulting in a poorer FCR (*p* < 0.001). This indicates that the reduced energy content in the diet negatively impacted the growth performance and feed efficiency of the lambs. Furthermore, the study revealed that the supplementation of RPC did not yield significant improvements in any of the measured performance parameters, including ADG, total BW, and FCR. Additionally, there were no significant interactions between the dietary RPC supplementation and the energy level of the diets in affecting these performance metrics (Table 2).

### 3.2. Determination of Serum Glucose and Lipid Profile

The impact of dietary RPC supplementation and energy level on the serum glucose concentration and lipid profile of Karayaka lambs is presented in Table 3. Dietary ME levels reduced serum glucose (*p* < 0.001) in lambs fed diets without or with RPC supplementation after day 42, but had no effect at other time points. There were no significant effects of RPC × ME on serum glucose. Neither dietary energy level nor RPC nor their interaction significantly affected serum lipid profiles. No significant effect of RPC × ME interaction was detected for serum TG, CHOL, HDL, or LDL levels.

### 3.3. Determination of Serum Protein Profile

The effect of dietary RPC supplementation and different energy levels on serum protein profile at various points throughout the experiment is shown in Table 4. The dietary treatments did not affect most of the serum protein profiles, including TP, ALB, GLB, the ALB/GLB ratio, and Cre. However, the main effect of RPC on serum urea-N levels was observed at 28 (*p* = 0.002) and 42 (*p* < 0.001) d. In addition, a significant interaction (*p* < 0.001) between RPC supplementation and dietary energy levels was observed at the end of the study.

### 3.4. Determination of Liver Function-Related Enzymes and Total Bilirubin

At various points throughout the experiment, dietary RPC or different ME levels did not significantly affect the levels of ALT, AST, ALP, and GGT enzymes involved in liver function in the blood serum of Karayaka lambs, as indicated in Table 5. Moreover, the TBIL level was not significantly affected in the serum of lambs fed RPC-supplemented diets containing all the ME levels studied.

### 3.5. Carcass Characteristics and Meat Quality

The results in Table 6 show that carcass characteristics (cold and hot carcass weights, cold and hot carcass yields, LD muscle area, and fat thickness over LD) in lambs fed a low-energy diet were lower (*p* < 0.001, *p* = 0.008, *p* = 0.004) than in lambs that received optimum dietary energy. The present study indicates that the main effect of dietary RPC is not significant on carcass characteristics. When considering meat quality parameters, including pH, color, water retention, cooking loss, and shear force (Table 6), the effect of dietary RPC × ME interaction and the effect of RPC or ME as the main effect were not significant for these parameters.

## 4. Discussion

Our experiment was designed to explore the hypothesis that dietary choline would enhance productive performance and meat quality by influencing energy and protein metabolism in lambs fed low-energy diets. The hypothesis was developed to reconcile observations that dietary RPC would be capable of sparing an energy matrix value of 250 kcal/kg (as reviewed by Tu et al. [24]). It is possible that the supplemental RPC leads to remarkable growth performance in the fattening lambs fed a low-energy diet through an interaction of diet energy and RPC supplementation. The data on BW, DMI, and serum glucose concentrations confirm that our model successfully induced low energy using 250 kcal/kg less energy than the optimum level.

DMI refers to the amount of DM consumed by animals, directly impacting their health and performance. The energy content of the diet also plays a crucial role in influencing nutrient intake, digestion, metabolic efficiency, and overall production performance [34]. In the present study, the DMI of lambs was significantly increased as the dietary ME decreased from 2750 to 25,000 kcal/kg, which is consistent with the results of Rios-Rincon et al. [35] and Jaborek et al. [36], who found that lambs fed low ME diets increased their DMI to compensate for energy deficiency. Furthermore, previous studies [8,30] reported the same results that dietary RPC had no effect on the DM intake of fattening lambs. In contrast, other studies have reported positive effects of RPC on feed intake. For example, Habeeb et al. [17] conducted a study on growing goats and observed a significant increase in feed intake following RPC supplementation. Similarly, Tu et al. [24] found that RPC had a stimulatory effect on feed intake in their study on growing goat nutrition. The effect of RPC supplementation on DMI could be influenced by many factors, such as choline purity, amount of RPC supplement offered, animal state, and rumen protection rate of choline [30]. It was found that RPC supplemented to the diet of dairy cows can be derived to trimethylamine via rumen microbial metabolism and further converted to trimethylamine N-oxide [37], while trimethylamine-N-oxide was associated with depressed DMI in some studies [38], but not in other studies [39]. Using RPC in the present study probably did not produce such an effect, as it was found to be similar to DMI between treatments. However, some previous studies have shown that RPC has the potential to increase appetite in ruminants [13,40], which was not observed in the current study. Therefore, the contrasting results observed in these studies highlight the need for further research to elucidate the effects of RPC on feed intake in lamb fattening.

A recent study demonstrated that RPC upregulated the expression of genes related to glucose and lipid metabolism, reducing body energy consumption in periparturient dairy cows [18]. Furthermore, a study by Caprarulo et al. [19] demonstrated that RPC supplementation at a maintenance energy level (1.40 Mcal NEL/kg) resulted in increased production compared to an excess energy level (1.63 Mcal NEL/kg). This was attributed to gene alterations related to hepatic glucose and lipid metabolism. Additionally, a previous study involving growing goats found that dietary RPC with low levels of dietary ME significantly altered growth performance [24]. It was proposed that betaine, an oxidizing product of choline, likely reduced the energy requirements for cellular maintenance, allowing more energy to be allocated toward animal growth [24]. Furthermore, Jin et al. [15] found that at optimal dietary ME levels (2954 kcal/kg), adequate RPC promoted thiamine absorption, sufficient thiamine suppressed urinary pyruvate levels by accelerating pyruvate metabolic fluxes via pyruvate dehydrogenase, thereby causing a sluggish energy metabolism, and ultimately a low-energy metabolism favored the weight gain of lambs. As shown in Table 2, the administration of RPC in this study could not maintain performance levels in lambs fed a low ME diet, probably due to the lack of the aforementioned effects on energy metabolism. Another possible explanation for the lack of response to RPC supplementation in this study is the presence of other methyl donors in the lamb diet and the absence of stress [41]. Indeed, the positive effect of dietary RPC on performance was observed in an experiment using a methionine-deficient diet of white Pekin ducks [42] and in stress-based experiments on male Zaraibi growing goats during the hot summer season [17]. These differences between studies may be attributed to several factors, such as the energy intake of the animals, the bioavailability of RPC, and the animal’s age, breed, and physiological status [13]. Similar to the results found in this study, previous studies did not observe improvements in growth performance when supplementing RPC in fattening lambs [8,14,26,30,43]. However, in a similar study by Li et al. [13], lambs with 0.25% RPC showed statistically higher ADG compared to animals with 0%, 0.5% and 0.75%. Our study differs from the study conducted by Li et al. [13] in several key aspects. Firstly, we utilized different levels of RPC supplementation. Additionally, our diets contained a higher percentage of protein (16% vs. 12%), potentially providing an excess of methyl donors for choline synthesis by rumen microorganisms [8]. Furthermore, our diets had a higher grain content and lower fiber content compared to those used by Li et al. [13], which could lead to alterations in the ruminal microbiota and, consequently, different effects on choline metabolism and other nutrients [8]. Another significant difference is the breed of the animals, an often-overlooked factor that has been shown to be associated with variations in rumen microbiome composition and feed efficiency in other ruminant species [8].

In the present study, all lambs gained BW. Consequently, the lambs were not metabolically stressed, and blood metabolites were generally within the normal range for livestock. In the present study, decreasing dietary ME levels did not affect serum lipid profiles but reduced glucose. This might be due to the lower ruminal propionate production, which is the only gluconeogenic volatile fatty acid in ruminants. Furthermore, in the current study, when lambs were fed the low ME diet, serum glucose was not improved in response to RPC. This is perhaps directly associated with no altered hepatic gluconeogenesis [44]. Similar to our results, previous studies [45,46] showed that RPC dietary supplementation did not affect the serum glucose indices of dairy cows [17,47]. In contrast to our findings, Tu et al. [24] reported that RPC supplementation in growing goats receiving low-energy diets increased glucose levels, probably due to a choline-mediated increase in glucose transport to provide energy for peptide synthesis. In lambs, it is observed that including herbal choline in the diet increases glucose concentration in blood serum [43,48]. These discrepancies could be attributed to many factors, such as the dose of choline, duration of supplementation, composition of the basal diets, and level of dietary energy.

Serum lipid levels were selected to be measured as an important indicator of lipid and energy metabolism [28]. Choline enhances phosphatidylcholine synthesis and may elevate HDL and LDL, as lipoprotein particles contain phospholipids, of which phosphatidylcholine accounts for 60–75% [49]. The present study found that the supplementation of RPC did not have any effect on serum lipid profiles, which is consistent with the results of Barcena-Gama et al. [26], who found that RPC supplementation did not affect serum TG, CHOL, LDL, and HDL levels. These findings are at odds with those of Li et al. [13], who observed that 0.50% RPC supplementation in lambs receiving 2507 kcal/kg ME resulted in a reduction in HDL concentration, while LDL concentration increased at 0.75% RPC. The reason for the discrepancy between our findings and the results of Li et al. [13] may be attributed to differences in the levels of RPC supplementation and breeds used in the two studies and the fact that RPC does not promote phosphatidylcholine synthesis. In numerous other studies, it was observed that the impact of RPC supplementation on TG, CHOL, LDL, and HDL levels exhibited considerable variability [8,16,17,30,43]. These variations may be associated with differences in animal breeds, diets, and physiological periods. In this study, the absence of any difference in serum lipid profile suggests that the energy level (2500 vs. 2750 kcal/kg) was not significantly low and that the body fat reserves of lambs in each diet group were similarly mobilized [50]. If lambs were at a higher risk of TG accumulation in their livers, there could be evidence to suggest that RPC may alter blood lipid profiles [51].

Blood protein profiles are an indirect nutritional indicator because they provide amino acids [52]. As a methyl-group donor, choline influences protein homeostasis by increasing synthesis and reducing breakdown [44]. However, in the present study, the inclusion of RPC in diets with different ME levels did not affect variables of protein profiles, including TP, ALB, GLB, and Cre, as observed by Rodriguez-Guerrero et al. [43] and Leal et al. [53]. A recent meta-analysis study reported that BioCholine doses over 8 g/d increased blood ALB, TP, and globulin and decreased serum urea-N [52]. The GLB values were inconsistent. Some experiments reported increases [26,53], while others reported reductions [48]. The concentration of serum urea-N reflects the efficiency of protein synthesis and the rate of urea recycling and is often used to assess the nutritional status of livestock [54]. An increase in serum urea-N may indicate an increase in protein catabolism and urea recycling in the liver, whereas a decrease may indicate an increase in protein synthesis [55]. In the current study, the effect of RPC on serum urea-N levels began to decrease after day 28 of the trial, and a significant interaction between RPC supplementation and dietary energy levels was revealed at the end of the trial. Specifically, the significant interaction at the end of the experiment indicates that the impact of RPC on serum urea-N levels was more pronounced when considering the combined effects of different energy levels and the duration of RPC administration. This interaction suggests a relationship whereby the efficacy of RPC in influencing serum urea-N levels was contingent on the dietary energy context. Zom et al. [56] described that choline functions as a methyl donor to spare methionine, ultimately increasing the use of methionine for protein synthesis. As a result, the modulation of nitrogen metabolism by choline seems to be an indirect mechanism that requires additional investigation. Similarly, Tu et al. [24] found that feeding 0.2% RPC to growing goats reduced serum urea-N, possibly due to different experimental conditions and the supplemented dose of RPC between the two studies. In contrast to our results, previous studies observed no change in serum urea-N levels in dairy cows supplemented with RPC [57,58]. 

The study did not find any effect of RPC supplementation on AST, ALT, ALP, and TBIL, which are important serum markers for the assessment of the health status of animals [59]. ALT and AST are transaminases found in animal tissue cells that are primarily involved in the transport of amino acids in the body and are concentrated in the liver. Serum ALT and AST activity is a crucial indicator of hepatocyte damage [60]. When liver cells are damaged or permeability increases, a large amount of ALT and AST will be released into the blood, which increases the activity of ALT and AST in the serum [61]. The results of liver function tests in this study are consistent with the findings of Habeeb et al. [17] and Tu et al. [24], who also did not observe any effect of RPC supplementation on plasma AST and ALT in growing goats. A recent study has shown that choline can be a safe and effective neuroprotective intervention against hyperbilirubinemia in choline-deficient mice [62]. The researchers reported that this effect might be because choline alters the rate of bilirubin biotransformation and mainly reduces serum-free bilirubin [62]. In addition, the dietary supplementation of RPC, especially in transition cows, improved hepatic function and decreased TBIL concentration, indicating a healthy liver status [63]. Therefore, we cannot exclude potent hepatoprotective effects provided by RPC because we used only indirect variables (i.e., liver enzyme levels). The absence of any significant changes in liver parameters due to choline supplementation in this study may be because a 250 kcal/kg energy deficit does not affect liver function [64]. As mentioned above, limiting the ME could have given different results and affected the clinical biochemical serum variables. 

Slaughter performance is one of the most critical indicators and can readily and effectively reflect the economic return of fattening lambs. Carcass weight and dressing percentage are key factors in determining slaughter performance [65]. Dietary energy levels are closely linked to carcass characteristics, with dressing percentage being a crucial indicator for measuring these traits [28]. In this study, decreasing dietary ME levels reduced carcass performance, which was in line with previous studies showing that the slaughter weight increased with increasing lamb live weight at slaughter [66,67]. In the current study, despite choline’s lipotropic effects and the known fat-reducing properties of its oxidized form (betaine) [68], fat thickness over LD and carcass characteristics did not show significant changes in response to RPC levels, as observed in previous studies [12,13,14,24,48]. However, Jahanian and Ashnagar [69] studied the lipotropic effects of adding choline to broiler diets. These authors reported an increased carcass yield and a reduction in abdominal fat in birds fed additional choline. The lack of improvement in final BW may explain why improvements in carcass traits were not seen with RPC supplementation. However, further research is needed to understand whether feeding choline in lamb fattening has similar metabolic benefits to betaine on carcass traits.

The pH level of meat is a crucial factor that impacts its quality. Alterations in pH levels can affect both the color and water retention of the meat [70]. Following slaughter, the pH level of meat decreases mainly due to anaerobic respiration in the body, which promotes the conversion of glycogen to lactic acid [71]. The function of glycogen in skeletal muscle is believed to be more affected by stress or the energy requirements of muscle rather than nutrition [72]. In this study, LD muscle pH was not affected by the addition of RPC to low or optimal ME diets, demonstrating that increasing or decreasing energy intake by 250 kcal/kg did not likely significantly affect muscle glycogen levels or cause oxidative stress. Tu et al. [24] reported similar results in growing goats, stating that there was no effect of RPC or ME on pH values measured 24 h after slaughter and no interaction effect of RPC × ME. Huo et al. [30] also reported that the supplementation of RPC to a pelleted total mixed ration did not improve the meat pH of fattening lambs. The color of meat is primarily determined by myoglobin, with higher concentrations of myoglobin resulting in a darker muscle color. As oxygen levels in the muscles decrease, oxymyoglobin is converted to ferric myoglobin, causing the muscle to darken [73]. The color of meat is a crucial indicator of product freshness, with consumers generally preferring light red or pink lamb meat [74]. Unlike L* and b*, a* has a stronger correlation with meat acceptance, and thus, consumers prefer meat with high a* and low L* values [75]. In this study, no effect of including RPC in the diet was observed on meat color properties (L*, a*, b*), but the color values of the LD muscle were found to be acceptable for consumers [76]. The lack of difference in the appearance of lamb meat in this study may be attributed to the fact that RPC supplementation at different energy levels did not affect postmortem lipid oxidation and intramuscular fat content [77]. Similar results were obtained by Tu et al. [24], who reported that RPC did not affect the L*, a*, and b* values of meat color. However, Martínez-García et al. [78] found that the L* value increased in lambs supplemented with RPC.

The water-holding capacity of meat is linked to the tenderness and juiciness of the meat [79]. After the death of an animal, protein degradation results in a reduction in water flow within the cells, resulting in a higher drip loss rate for meat that loses nutrients more rapidly [80]. The water-holding capacity of meat is minimally affected by the energy content of the diet, according to Zhang et al. [65]. Choline is necessary for the synthesis of phospholipids, which serve as an integral part of biological membranes. Therefore, choline plays a vital role in the fluidity and integrity of cell membranes, which can limit the formation of free radicals resulting from lipid oxidation to some extent and alleviate the oxidation reaction, thus reducing sarcoplasmic fluid leakage and increasing meat tenderness and water-holding capacity [81]. Li et al. [13] proposed that a diet containing 0.25% RPC can increase the water-holding capacity and reduce the drip loss and the shear force through increasing phosphatidylcholine synthesis via choline. However, in the present study, RPC and the energy content of the diet have not improved the water-holding capacity of LD meat muscle. This situation may be attributed to RPC not causing an increase in phosphatidylcholine synthesis, thereby maintaining cell integrity unchanged. Shear force is negatively correlated with tenderness [82] and increases with age [83] but is minimally affected by dietary treatment [65]. In this study, the shear force was not affected by energy levels and RPC. Similar to the current study, adding RPC at different energy levels in goat diets was found to have no significant effect on cooking loss and shear force [24]. In contrast to this study, Huo et al. [30] found that drip loss was 65% higher in the group supplemented with 5 g/kg choline compared to the control group. Conversely, some studies have suggested that RPC added to the diet can increase the water-holding capacity of muscles by oxidizing betaine [84,85] and drip loss [30].

This study represents the first comparative analysis of production performance, meat characteristics and blood parameters in lambs fed diets supplemented with RPC at different ME levels, but it has limitations. Firstly, the sample size of the trial lambs is limited. Secondly, accurately describing the process based on energy-lipid, protein, and liver function tests is challenging. Future studies should take these limitations into account.

## 5. Conclusions

In conclusion, the supplementation of RPC at 0 and 4 g/kg did not affect lamb growth performance and carcass characteristics. Given the lack of significant improvements in performance metrics, the RPC supplementation would not result in net cost savings. On the contrary, the cost of including RPC in the diet would represent an additional expense without corresponding performance benefits. The results of the current study indicate that there is no benefit to be gained from the supplementation of RPC in the low-energy diet of fattening lambs. However, it may play a role in modulating nitrogen metabolism under different dietary energy conditions, as indicated by the significant reduction in serum urea-N levels. Further research could examine whether the impact of RPC on nitrogen metabolism could be more pronounced, particularly in animals exposed to varying energy levels.

## Figures and Tables

**Table 1:** Ingredients and nutrient composition of the used concentrate feeds and alfalfa hay.

Ingredient, % of DM	Optimum-Energy	Low-Energy	Alfalfa Hay
Barley	40.00	39.10	-
Corn	26.47	10.00	-
Wheat cracked	7.45	2.00	-
Sunflower meal	11.02	22.08	-
Cottonseed meal	2.00	2.00	-
Soybean meal	6.96	-	-
Wheat bran	2.00	10.72	-
Wheat bonkalite	-	6.00	-
Molasses	2.00	6.00	-
Marble powder	1.50	1.50	-
Salt	0.40	0.4	-
Sodium bicarbonate	0.10	0.10	-
Vitamin (Premix) *	0.10	0.10	-
Nutrient composition, % of DM			
Dry matter	87.62	87.24	88.00
Crude protein	16.00	16.00	16.08
Crude cellulose	5.96	9.42	28.84
Ether extract	3.20	2.63	1.76
Metabolizable energy (ME) ^†^, kcal/kg	2750	2500	1950

* 1 kg of Vitamin Premix contains (5000 IU vitamin A; 1000 IU vitamin D3; 5 mg vitamin E; 50 mg manganese; 50 mg zinc; 10 mg copper; 50 mg magnesium; 0.15 mg cobalt; 0.30 mg iodine; 0.15 mg selenium; 450 mg phosphorus; 620 mg calcium and 90 mg sodium carbonate. ^†^ Calculated based on tabular value of NRC (2007) [3].

**Table 2:** Effects of supplemental RPC on the growth performance of lambs fed two levels of dietary ME (*n* = 7).

Item	Day	−RPC ^†^		+RPC ^†^		SEM ^†^	*p* Value		
		Optimum ME ^†^	Low ME ^†^	Optimum ME ^†^	Low ME ^†^		RPC	ME	RPC × ME
Body weight (kg)	Initial BW ^‡^, kg	26.92	26.89	26.74	26.84	0.269	0.843	0.951	0.911
	d 14	30.10	30.01	30.15	29.95	0.260	0.995	0.793	0.923
	d 28	33.97	33.25	33.79	33.42	0.274	1.000	0.350	0.757
	d 42	37.63	35.99	37.62	36.28	0.297	0.797	0.012	0.797
	d 56	41.07	39.35	41.35	39.61	0.291	0.596	<0.001	0.978
ADG ^‡^ (g/day)	d 1–14	227.55	222.96	243.87	222.45	4.156	0.337	0.120	0.307
	d 15–28	276.02	231.12	25.69	247.96	5.062	0.976	0.003	0.060
	d 29–42	261.73	195.91	273.98	204.08	8.483	0.375	<0.001	0.858
	d 43–56	245.40	240.30	266.32	237.75	7.866	0.572	0.304	0.472
	Overall	252.68	222.57	260.97	228.05	3.769	0.142	<0.001	0.759
DMI ^‡^ (kg/days)	d 1–14	1.11	1.09	1.17	1.10	0.012	0.119	0.075	0.385
	d 15–28	1.31	1.28	1.30	1.37	0.015	0.522	0.206	0.118
	d 29–42	1.24	1.33	1.26	1.36	0.012	0.199	<0.001	0.629
	d 43–56	1.25	1.37	1.32	1.40	0.020	0.239	0.011	0.539
	Overall	1.22	1.30	1.25	1.31	0.009	0.067	<0.001	0.503
FCR ^‡^	d 1–14	4.89	5.02	5.10	4.95	0.061	0.600	0.955	0.279
	d 15–28	4.77	5.65	4.96	5.35	0.099	0.718	<0.001	0.141
	d 29–42	4.73	6.78	4.60	6.66	0.221	0.544	<0.001	0.976
	d 43–56	5.09	5.73	4.95	5.88	0.126	0.987	0.001	0.519
	Overall	4.70	5.84	4.78	5.74	0.128	0.953	<0.001	0.605

^†^ SEM, standard error of the mean; −RPC, without RPC; +RPC: 4 g RPC/head/day; Optimum ME, 2750 kcal ME/kg; Low ME, 2500 kcal ME/kg; ^‡^ DMI, dry matter intake; BW, body weight; ADG, average daily gain; FCR, feed conversion ratio.

**Table 3:** Effects of RPC supplementation and energy levels on blood serum glucose and lipid profiles of Karayaka lambs at various points throughout the experiment (*n* = 7).

Item	Day	−RPC ^†^		+RPC ^†^		SEM ^†^	*p* Value		
		Optimum ME ^†^	Low ME ^†^	Optimum ME ^†^	Low ME ^†^		RPC	ME	RPC × ME
Glucose (mg/dL)	0 d	62.28	63.71	64.85	64.00	0.432	0.101	0.736	0.185
	14 d	67.43	67.64	68.49	66.95	0.359	0.208	0.377	0.251
	28 d	67.57	69.00	67.71	66.42	0.666	0.382	0.959	0.330
	42 d	68.27	65.23	68.49	64.65	0.474	0.754	<0.001	0.498
	56 d	68.10	66.00	68.49	65.69	0.380	0.943	<0.001	0.542
Triglyceride (mg/dL)	0 d	11.28	11.00	11.85	11.71	0.232	0.187	0.655	0.881
	14 d	14.52	12.70	13.52	13.65	0.409	0.981	0.327	0.257
	28 d	13.28	13.85	12.42	12.57	0.403	0.205	0.668	0.797
	42 d	15.12	13.41	12.95	12.58	0.614	0.246	0.417	0.600
	56 d	13.57	12.71	14.24	13.28	0.535	0.898	0.376	0.525
Cholesterol (mg/dL)	0 d	42.98	42.92	43.61	44.44	1.024	0.623	0.860	0.838
	14 d	43.86	44.65	42.15	43.11	0.504	0.123	0,396	0.932
	28 d	43.11	42.25	44.31	42.21	1.079	0.801	0.518	0.788
	42 d	43.08	42.53	42.27	41.90	0.325	0.304	0.506	0.890
	56 d	43.17	41.73	42.93	42.09	0.973	0.887	0.584	0.976
HDL ^‡^ (mg/dL)	0 d	25.64	25.54	26.14	27.95	0.461	0.116	0.347	0.295
	14 d	26.83	27.86	27.14	26.94	0.316	0.652	0.542	0.365
	28 d	27.04	27.55	25.98	27.08	0.892	0.689	0.672	0.878
	42 d	26.98	26.96	27.49	27.75	0.342	0.380	0.859	0.857
	56 d	26.60	27.47	24.44	28.75	0.783	0.272	0.104	0.779
LDL ^‡^ (mg/dL)	0 d	7.14	7.28	9.57	8.57	0.441	0.038	0.616	0.505
	14 d	11.42	9.90	10.19	12.96	0.518	0.615	0.859	0.107
	28 d	10.00	9.14	9.85	10.14	0.386	0.599	0.725	0.484
	42 d	11.19	10.28	12.43	9.52	0.816	0.890	0.276	0.563
	56 d	9.14	9.28	10.28	11.00	0.654	0.835	0.754	0.302

^†^ SEM, standard error of the mean; −RPC, without RPC; +RPC: 4 g RPC/head/day; Optimum ME, 2750 kcal ME/kg; Low ME, 2500 kcal ME/kg; ^‡^ HDL, high-density lipoprotein cholesterol; LDL, low-density lipoprotein cholesterol.

**Table 4:** Effects of RPC supplementation and energy levels on serum protein profiles of Karayaka lambs at various points throughout the experiment (*n* = 7).

Item	Day	−RPC ^†^		+RPC ^†^		SEM ^†^	*p* Value		
		Optimum ME ^†^	Low ME ^†^	Optimum ME ^†^	Low ME ^†^		RPC	ME	RPC × ME
Total protein (g/dL)	0 d	6.35	6.32	6.51	6.47	0.050	0.157	0.731	0.945
	14 d	6.48	7.33	6.93	6.78	0.405	0.954	0.694	0.573
	28 d	6.27	6.52	6.50	6.34	0.076	0.893	0.753	0.200
	42 d	6.42	7.09	7.39	6.86	0.388	0.659	0.932	0.447
	56 d	6.47	6.50	6.35	6,.0	0.073	0.781	0.926	0.313
Albumin (A) (g/dL)	0 d	3.11	3.25	3.24	3.15	0.032	0.829	0.666	0.094
	14 d	2.87	3.87	3.87	3.46	0.285	0.652	0.594	0.265
	28 d	2.98	3.25	3.25	3.21	0.045	0.215	0.215	0.093
	42 d	3.74	3.44	3.28	3.15	0.222	0.444	0.659	0.856
	56 d	3.02	3.30	2.70	2.94	0.115	0.951	0.274	0.148
Globulin (G) (mg/dL)	0 d	3.30	3.15	3.41	3.37	0.046	0.085	0.319	0.589
	14 d	3.61	3.46	3.11	3.32	0.439	0.741	0.976	0.853
	28 d	3.27	3.22	3.22	3.18	0.086	0.818	0.818	1.000
	42 d	2.68	3.65	4.11	3.71	0.466	0.460	0.775	0.493
	56 d	3.42	3.20	3.22	3.37	0.067	0.187	0.757	0.918
A/G ^‡^	0 d	0.94	1.03	0.95	0.94	0.014	0.151	0.180	0.094
	14 d	0.98	2.21	1.83	1.41	0.301	0.969	0.520	0.200
	28 d	0.93	1.06	1.02	1.01	0.038	0.804	0.448	0.388
	42 d	1.52	1.52	1.24	1.75	0.273	0.967	0.669	0.674
	56 d	0.89	1.03	0.84	0.87	0.039	0.473	0.291	0.177
Serum urea-N (mg/dL)	0 d	22.32	23.75	22.64	22.12	0.294	0.280	0.449	0.115
	14 d	22.94	22.35	22.15	21.88	0.398	0.466	0.619	0.857
	28 d	21.72	22.25	19.07	20.44	0.383	0.002	0.151	0.517
	42 d	23.14	24.06	19.64	20.03	0.560	<0.001	0.430	0.778
	56 d	23.82 ^a^	21.32 ^ab^	17.42 ^c^	20.45 ^b^	0.560	0.001	0.723	<0.001
Creatinine (mg/dL)	0 d	0.50	0.53	0.56	0.55	0.008	0.21	0.521	0.253
	14 d	0.50	0.42	0.42	0.53	0.038	0.855	0.932	0.256
	28 d	0.56	0.61	0.57	0.57	0.011	0.531	0.350	0.239
	42 d	0.61	0.64	0.51	0.64	0.032	0.434	0.270	0.434
	56 d	0.65	0.73	0.71	0.73	0.182	0.342	0.198	0.382

^†^ SEM, standard error of the mean; −RPC, without RPC; +RPC: 4 g RPC/head/day; Optimum ME, 2750 kcal ME/kg; Low ME, 2500 kcal ME/kg; ^‡^ A/G, albumin/globulin ratio. ^a–c^ Means with different letters superscripts at the same row differ significantly at (*p* < 0.05).

**Table 5:** Effects of RPC supplementation and energy levels on liver function of Karayaka lambs at various points throughout the experiment (*n* = 7).

Item	Day	−RPC ^†^		+RPC ^†^		SEM ^†^	*p* Value		
		Optimum ME ^†^	Low ME ^†^	Optimum ME ^†^	Low ME ^†^		RPC	ME	RPC × ME
ALT ^‡^ (U/L)	0 d	18.14	17.57	21.00	17.42	0.455	0.890	0.120	0.620
	14 d	18.84	18.67	18.89	18.14	0.283	0.633	0.367	0.574
	28 d	17.14	17.71	17.28	17.28	0.345	0.846	0.698	0.698
	42 d	18.70	18.51	19.11	18.32	0.416	0.899	0.592	0.742
	56 d	18.28	18.28	17.85	20.28	0.398	0.120	0.120	0.307
AST ^‡^ (U/L)	0 d	80.85	71.42	81.28	71.00	1.304	1.000	<0.001	0.823
	14 d	82.72	81.60	83.22	82.37	0.418	0.472	0.272	0.878
	28 d	81.14	81.71	78.71	77.25	1.740	0.353	0.907	0.785
	42 d	82.07	82.28	82.72	81.98	0.429	0.853	0.777	0.615
	56 d	81.14	80.57	78.74	84.28	1.553	0.345	0.441	0.842
ALP ^‡^ (U/L)	0 d	178.00	176.85	191.28	191.14	6.515	0.318	0.963	0.971
	14 d	179.40	178.00	179.20	179.60	0.582	0.577	0.690	0.475
	28 d	178.00	176.85	191.28	191.14	6.515	0.318	0.963	0.971
	42 d	226.20	225.20	228.00	226.40	0.634	0.227	0.228	0.757
	56 d	234.00	214.71	213.71	245.56	7.585	0.104	0.681	0.730
AST/ALP ^‡^	0 d	0.56	0.46	0.55	0.47	0.012	0.852	<0.001	0.527
	14 d	0.46	0.45	0.46	0.45	0.002	0.862	0.390	0.862
	28 d	0.47	0.46	0.45	0.42	0.025	0.598	0.654	0.832
	42 d	0.36	0.36	0.36	0.36	0.001	0.644	1.000	0.664
	56 d	0.42	0.43	0.37	0.38	0.016	1.000	0.731	0.103
GGT ^‡^ (U/L)	0 d	92.28	72.14	92.14	79.57	2.557	0.387	0.001	0.369
	14 d	77.60	76.60	76.00	74.40	0.715	0.208	0.382	0.838
	28 d	77.00	73.57	75.28	78.00	0.992	0.502	0.859	0.136
	42 d	77.20	77.40	76.40	77.60	0.612	0.822	0.602	0.709
	56 d	77.00	78.00	75.28	73.57	0.992	0.502	0.859	0.136
TBIL ^‡^ (mg/dL)	0 d	0.42	0.32	0.44	0.42	0.018	0.111	0.111	0.226
	14 d	0.30	0.30	0.31	0.30	0.007	0.684	0.602	0.602
	28 d	0.30	0.27	0.32	0.28	0.014	0.490	0.254	0.817
	42 d	0.31	0.32	0.32	0.33	0.006	0.892	0.588	0.786
	56 d	0.32	0.35	0.35	0.35	0.222	0.774	0.774	0.774

^†^ SEM, standard error of the mean; −RPC, without RPC; +RPC: 4 g RPC/head/day; Optimum ME, 2750 kcal ME/kg; Low ME, 2500 kcal ME/kg; ^‡^ ALP, alkaline phosphatase; ALT, alanine transaminase; AST, aspartate transaminase; AST/ALP, aspartate transaminase/alanine transaminase ratio; TBIL, total bilirubin; GGT, g-glutamyl transpeptidase.

**Table 6:** Effects of supplemental RPC on carcass characteristics and meat quality of lambs fed two levels of dietary ME (*n* = 7).

	−RPC ^†^		+RPC ^†^		SEM ^†^	*p* Value		
	Optimum ME ^†^	Low ME ^†^	Optimum ME ^†^	Low ME ^†^		RPC	ME	RPC × ME
Carcass performance								
Hot carcass weight (kg)	19.32	17.57	19.74	17.71	0.211	0.785	<0.001	0.166
Cold carcass weight (kg)	18.57	17.28	18.98	17.00	0.192	0.767	<0.001	0.140
Hot dressing percentage (%)	47.04	45.45	47.72	44.38	0.347	0.703	<0.001	0.097
Cold dressing percentage (%)	45.20	43.99	45.89	42.94	0.307	0.899	<0.001	0.388
Longissumus dorsi area (cm^2^)	11.96	10.70	11.66	10.64	0.222	0.592	0.008	0.719
Fat thickness over LD (mm)	3.95	3.54	3.82	3.25	0.097	0.125	0.004	0.532
Meat quality								
pH_24h_	5.76	5.79	5.74	5.82	0.637	0.932	0.152	0.556
Color characteristics for LD_24h_								
L* (Lightness)	43.06	45.20	44.54	45.56	0.390	0.176	0.034	0.391
a* (Redness)	17.95	17.44	17.47	18.30	0.368	0.824	0.851	0.446
b* (Yellowness)	12.62	12.88	12.20	12.89	0.206	0.655	0.310	0.634
Water-holding capacity (%)	38.31	38.29	39.03	38.57	0.353	0.545	0.772	0.791
Drip loss (%)	2.84	2.69	2.74	2.82	0.038	0.820	0.666	0.179
Cooking loss (%)	27.46	28.01	27.91	27.73	0.231	0.905	0.935	0.348
Shear force (kg/cm^2^)	4.85	4.96	4.81	5.25	0.195	0.784	0.541	0.718

^†^ SEM, standard error of the mean; −RPC, without RPC; +RPC: 4 g RPC/head/day; Optimum ME, 2750 kcal ME/kg; Low ME, 2500 kcal ME/kg.

## Data Availability

The data will be provided upon request from the authors.
